# Synergistic activities of colistin combined with other antimicrobial agents against colistin-resistant *Acinetobacter baumannii* clinical isolates

**DOI:** 10.1371/journal.pone.0270908

**Published:** 2022-07-13

**Authors:** Mashal M. Almutairi

**Affiliations:** Department of Pharmacology and Toxicology, College of Pharmacy, King Saud University, Riyadh, Saudi Arabia; Foshan University, CHINA

## Abstract

Emerging resistance to colistin in *Acinetobacter baumannii* clinical strains is concerning because of the limited therapeutic choices for these important clinical pathogens. We studied the *in vitro* activities of different colistin-based antimicrobial agent combinations against colistin-resistant *Acinetobacter baumannii*. Fourteen clinical isolates of colistin-resistant *Acinetobacter baumannii* were obtained between 2015 and 2016. To identify colistin-based combinations with synergistic activities, multiple two antimicrobial combinations based on 8 commercially available drugs were evaluated by the checkerboard method. The most effective colistin-based combinations were vancomycin, aztreonam, ceftazidime and imipenem which showed synergistic activities against all examined strains. Colistin-rifampin showed synergy against four strains. Colistin-tigecycline and colistin-amikacin mostly showed indifferent results. By using the checkerboard tests, we were able to find the most promising colistin-based combinations that may provide more therapeutic options against colistin-resistant *Acinetobacter baumannii*.

## Introduction

*Acinetobacter baumannii* is a Gram-negative opportunistic pathogen and is considered one of the most important nosocomial pathogens and can cause nosocomial diseases such as pneumonia, septicaemia, urinary tract infections, endocarditis, and meningitis [1, 2]. *A*. *baumannii* has become more challenging due to its ability to develop resistance to a wide spectrum of antimicrobial agents, including carbapenems [3]. Today, there are different *A*. *baumannii* strains with various resistance mechanisms which include multidrug-resistant *A*. *baumannii* (MDR-AB), carbapenem-resistant *A*. *baumannii* (CRAB), and colistin-resistant *A*. *baumannii* (CoR-AB). These strains are associated with prolonged hospitalisations and high mortality and morbidity rates [4, 5]. *A*. *baumannii* has resistance mechanisms that include the production of beta-lactamases and changes in the outer membrane proteins against beta-lactams, drug-inactivating enzymes, target mutation and efflux pump against aminoglycosides [6].

Colistin is considered among the most powerful antimicrobials against multidrug-resistant (MDR) Gram-negative bacteria *in vitro* and it has been reintroduced to clinical practice for the treatment of carbapenem-resistant *A*. *baumannii* [3]. However, colistin-resistant *A*. *baumannii* strains have been documented worldwide [7, 8]. These strains are not only resistant to colistin but also to most antimicrobial agents, which makes the treatment options severely limited [3, 8]. To further complicate the situation, colistin therapy is frequently discontinued because of colistin-associated nephrotoxicity which happens in about 40% of patients [8–10]. To provide solutions for the previous problems, the activities of colistin with other antimicrobial agents against colistin-resistant *A*. *baumannii* strains should be investigated. The colistin-based combinations will serve to: (i) overcome the resistance against colistin and other antimicrobial agents; (ii) increase the combination efficacy over monotherapy; and (iii) decrease colistin-associated toxicities. Previous studies have shown the existence of synergistic interactions when colistin is combined with antimicrobials against various Gram-negative clinical isolates [11–13]. However, to the best of our knowledge, there is no such study for colistin-based combination against Gram-negative clinical isolates from Saudi Arabia. We hypothesize that by combining other antimicrobial agents with colistin, the colistin-based resistance can be overcome by different mechanisms of synergy. The main objective of the study is to evaluate the *in vitro* efficacy of colistin-based antimicrobial agent combinations, using 8 clinically approved antimicrobial agents against colistin-resistant *A*. *baumannii* clinical isolates using the checkerboard method.

## Materials and methods

### Clinical isolates and antimicrobial agent selection

Patients infected with colistin-resistant *A*. *baumannii* were identified at Ad-Dawadmi, Saudi Arabia, between 2015 and 2016. The susceptibility testing of antimicrobial agents was done on all clinical isolates based on the orders of the treating physician. In our study, 14 representative colistin-resistant *A*. *baumannii* isolates from 14 different patients were collected and identified by a Vitek 2 system (bioMérieux Inc., La Balme Les Grottes, France). The following 8 antimicrobial agents were chosen because of their activities against MDR *A*. *baumannii*: colistin, tigecycline, vancomycin, rifampin, imipenem, amikacin, aztreonam, and ceftazidime [2, 14–16]. All clinical strains were a gift from Dr Zeyad Alzeyadi (Ethics Research Committee Approval, Shaqra University ERC No. ERC_SU_20210078) [17]. All antimicrobial agent powders were purchased from Toronto Research Chemicals, Toronto, Canada.

### Susceptibility examination and interpretation

All *in vitro* antimicrobial susceptibility tests were done in triplicate. We used the broth microdilution technique following Clinical and Laboratory Standards Institute (CLSI) guidelines [14]. Mueller-Hinton broth was used for all susceptibility testing. CLSI susceptibility criteria were used, except with aztreonam, vancomycin, tigecycline and rifampin. A minimum inhibitory concentration (MIC) of colistin of ≥4 mg/l was considered as resistance [18]. There are no susceptibility breakpoints available for rifampin and tigecycline within the CLSI guidelines. Therefore, CLSI criteria for *staphylococci* were used with rifampin (MIC ≥4 mg/l is considered resistance). For tigecycline, criteria for *Enterobacteriaceae* were used (MIC >2 mg/l as resistance) based on the European Committee on Antimicrobial Susceptibility Testing (EUCAST) [19]. *Escherichia coli* ATCC 25922 was used for quality control purposes, and all the obtained results were within the CLSI quality control recommendations [18].

### Colistin combinations synergy testing by the checkerboard method

To identify synergistic activities, the checkerboard method was used in 96-well plates containing colistin with 1 of 7 other antimicrobial agents. Each antimicrobial was diluted using the two-dilution method, with testing concentrations in the range of 0.004X MIC to 2-4X MIC. After the addition of the antimicrobials, the bacteria were added with an initial inoculum of approximately 5x10^5^ CFU/ml. All 96-well plates were incubated at 37°C for 48 hours under aerobic conditions [20]. After incubation, wells that display turbidity were indicated to have bacterial growth. We calculated the fractional inhibitory concentration index (FICI) for each antimicrobial for each combination. After that, the means of the FICI of all non-turbid wells located along the turbidity/non-turbidity interface were calculated [21]. The FICI values for each antimicrobial combination against each clinical strain were interpreted according to the following metric: FICI of ≤ 0.5 was considered to be synergism; FICI between 0.5 and 1 was considered to have partial synergism; FICI of ≥1 but <4 was indicated as indifference; FICI of ≥4 is indicated as antagonism [11, 14, 21]. A checkerboard method was performed in triplicate format for each experiment.

## Results

### Microbiological features of colistin-resistant *A*. *baumannii*

We collected 14 colistin-resistant *A*. *baumannii* strains (8 sputum isolates, 3 wound isolates, 1 isolate from an arterial line, 1 from urine and 1 from ascites fluid; see Table 1). The results of the MICs of antimicrobial agents against each isolate are summarised in Table 2. In contrast with Vitek’s results, not all strains were found resistant to colistin. Strains a, b, c, e, f, h, i, l and n had MIC values <0.25–2 mg/ml which was less than the CLSI breakpoint. The remaining 5 strains were considered resistant to colistin with MIC for each of 1024 mg/ml. Therefore, these 5 strains were used to investigate colistin synergistic activities with other antimicrobial agents. They were resistant to colistin, vancomycin, imipenem, amikacin, aztreonam and ceftazidime. They varied in their susceptibilities to tigecycline and rifampin: d, g and m strains were susceptible to tigecycline and rifampin; j was susceptible to rifampin but resistant to tigecycline; and k strain was susceptible to tigecycline but resistant to rifampin.

**Table 1:** Distribution of *A*. *baumannii* isolates based on sample type.

A. baumannii Strain	Sample Type
a	Ascites fluid
b	Sputum
c	Sputum
d	Sputum
e	Wound
f	Sputum
g	Arterial line
h	Wound
i	Sputum
j	Urine
k	Sputum
l	Sputum
m	Wound
n	Sputum

**Table 2:** Antimicrobial agents MIC with colistin-resistant *A*. *baumannii* clinical isolates.

Antimicrobial MIC (mg/ml)^a^								
Strain	CST	TGC	VAN	RIF	IMP	AMK	ATM	CAZ
*E*. *coli* ATCC 25922	0.5	0.5	>256	8	0.5	8	<0.125	<0.25
a	1	2	4096	16	8	256	256	256
b	0.5	1	512	2	64	16	256	1024
c	<0.25	1	256	4	32	16	512	1024
d	1024	1	256	2	64	4096	128	512
e	<0.25	1	256	4	32, 64	8	512	1024
f	0.5	1	512	4	32, 64	16	1024	1024
g	1024	1	256	4	64	>4096	256	512
h	<0.25	1	1024	4	32, 128	32	1024	1024
i	2	2	4096	32	1, 4	4	8	1
j	1024	4	2048	1	64	>4096	64	256
k	1024	2	512	32	32	4096	256	512
l	2	1	256	4	64	16	1024	1024
m	1024	1	256	2	64	>4096	256	512
n	2	1	256	4	64	8	512	1024

^a^ Abbreviations: CST, colistin; TGC, tigecycline; VAN, vancomycin; RIF, rifampin; IMP, Imipenem AMK, amikacin; ATM, aztreonam; CAZ, ceftazidime. CST resistant strains are highlighted.

### Checkerboard synergy test

Checkerboard tests were performed to investigate the presence of synergistic interactions between colistin and other antimicrobials against the colistin-resistant *A*. *baumannii*. As shown in Table 3, the colistin-vancomycin combination (5/5, 100%) was fully synergistic against all tested *A*. *baumannii* strains. The colistin-rifampin combination was synergistic against 4 of the 5 tested strains. The combinations of colistin-imipenem, colistin-ceftazidime and colistin-aztreonam showed either synergy or partial synergy against all tested strains. Colistin-tigecycline and colistin-amikacin showed indifference against 4 and 5 strains, respectively. Fortunately, no antagonistic interactions were seen with all examined antimicrobials combinations. Our results showed that the majority of colistin-based synergistic interactions occurred with vancomycin, rifampin, ceftazidime and aztreonam where previous studies showed the majority of colistin positive reactions happened only with vancomycin and rifampin [22].

**Table 3:** The checkerboard test of colistin-resistant *A*. *baumannii*.

Isolate(s) with the test result			
Antimicrobial Agent Combinations	Synergistic (FICI ≤0.5)	Partially synergistic (0.5 <FICI < 1)	Indifferent (1 ≤FICI < 4)
CST + TGC	-	j	d, g, k, m
CST + VAN	d, g, j, k, m	-	-
CST + RIF	d, g, k, m	-	j
CST + IMP	g, j	d, k, m	-
CST + AMK	-	-	d, g, j, k, m
CST + ATM	d, g, k, m	j	-
CST + CAZ	d, g, j, k	m	-

## Discussion

The main objective of the study was to evaluate the *in vitro* synergistic activities of colistin-based antimicrobial combinations against colistin-resistant *A*. *baumannii*. The commonly used antimicrobial agent combinations were tested by the checkerboard method. Colistin-rifampin and colistin-cell wall inhibitors (vancomycin, aztreonam, imipenem and ceftazidime) combinations showed synergistic activities against most isolates as indicated by the checkerboard method.

Colistin-resistant *A*. *baumannii* may become resistant to colistin due to modifications of the outer membrane which may increase the permeability to other cell wall antimicrobial agents. Previous studies have reported that colistin-resistant *A*. *baumannii* strains were more susceptible to other antimicrobial agents than colistin-susceptible strains [23]. Antimicrobial agents showed high MICs against colistin-resistant strains in our study and another recent study [8]. These variations were most likely because of concurrent exposure to different antimicrobial agents.

The most studied combination *in vitro* is colistin with rifampin [24]. Our study showed a strong synergistic activity for colistin when combined with rifampin. This combination was found to be synergistic (FICI≤0.5) against 4 of the 5 *A*. *baumannii* strains. Therefore, the clinical efficacy of the colistin-rifampin combination should be further examined in colistin-resistant *A*. *baumannii* infections.

Vancomycin showed high MICs against our isolates. We proposed that vancomycin might be synergistic with colistin regardless of its high MIC, due to colistin’s effect on the *A*. *baumannii* outer membrane. Vancomycin consistently showed synergistic interactions when combined with colistin, in alignment with earlier *in vitro* and *in vivo* studies [25, 26]. In our study, other cell wall inhibitors agents such as ceftazidime, aztreonam, and imipenem showed synergistic activities. Previous studies showed that the majority of colistin based synergistic interactions occurred with vancomycin and rifampin [22]. These difference in the synergistic interaction patterns between our work and others may be because of the difference on the genetic markup of the tested strains. Therefore, it is very crucial to study the antimicrobial synergistic patterns in different geographical locations to account for the genetic variations between the clinical isolates of *A*. *baumannii*.

Tigecycline is considered an effective therapeutic choice for MDR *A*. *baumannii*. In this study, tigecycline showed low MIC against colistin-resistant strains. However, colistin-tigecycline combinations failed to show any synergistic activities against any of the strains. This may be because tigecycline targets the bacterial ribosome and unaffected by the disruption of the cell wall by colistin. Amikacin showed high MIC values and failed to give any synergistic activities with colistin against all tested strains.

The study has some limitations. The colistin-resistant bacteria were collected from one site and the exact colistin-based resistance mechanisms were not determined by genotyping. We performed an *in vitro* synergistic study which cannot test for clinical outcomes. We believe *in vivo* animal and clinical studies are required to examine the clinical outcomes where the colistin based synergistic interactions with other antimicrobial agents can be confirmed. However, the obtained results can provide a guide for the clinicians to select the best colistin combinations against clinical MDR *A*. *baumannii*.

In conclusion, by using the checkerboard method, we found that colistin-based combinations with rifampin, vancomycin, or β-lactams have synergistic activities. Therefore, these combinations might provide therapeutic benefits against colistin-resistant *A*. *baumannii* infections.

## Supporting information

S1 Table The checkerboard results for the tested clinical strains with colistin combined with other antimicrobials. (I) CST was combined with TGC (a), VAN (b), RIF (c), IMP (d), AMK (e), ATM (f) or CAZ (g) against strain d. (II) CST was combined with TGC (a), VAN (b), RIF (c), IMP (d), AMK (e), ATM (f) or CAZ (g) against strain g. (III) CST was combined with TGC (a), VAN (b), RIF (c), IMP (d), AMK (e), ATM (f) or CAZ (g) against strain j. (IV) CST was combined with TGC (a), VAN (b), RIF (c), IMP (d), AMK (e), ATM (f) or CAZ (g) against strain k. (V) CST was combined with TGC (a), VAN (b), RIF (c), IMP (d), AMK (e), ATM (f) or CAZ (g) against strain m.
(DOCX)
