# Supplementary material for: Synergistic activities of colistin combined with other antimicrobial agents against colistin-resistant Acinetobacter baumannii clinical isolates
Source: PLoS One. 2022 Jul 13;17(7):e0270908. doi: 10.1371/journal.pone.0270908 (PMC9278772; doi:10.1371/journal.pone.0270908)
Supplement: S1 Table — (I) CST was combined with TGC (a), VAN (b), RIF (c), IMP (d), AMK (e), ATM (f) or CAZ (g) against strain d. (II) CST was combined with TGC (a), VAN (b), RIF (c), IMP (d), AMK (e), ATM (f) or CAZ (g) against strain g. (III) CST was combined with TGC (a), VAN (b), RIF (c), IMP (d), AMK (e), ATM (f) or CAZ (g) against strain j. (IV) CST was combined with TGC (a), VAN (b), RIF (c), IMP (d), AMK (e), ATM (f) or CAZ (g) against strain k. (V) CST was combined with TGC (a), VAN (b), RIF (c), IMP (d), AMK (e), ATM (f) or CAZ (g) against strain m. (DOCX) [file pone.0270908.s001.docx]

**S1 Table: The checkerboard results for the tested clinical strains with colistin combined with other antimicrobials.** (I) CST was combined with TGC (a), VAN (b), RIF (c), IMP (d), AMK (e), ATM (f) or CAZ (g) against strain d. (II) CST was combined with TGC (a), VAN (b), RIF (c), IMP (d), AMK (e), ATM (f) or CAZ (g) against strain g. (III) CST was combined with TGC (a), VAN (b), RIF (c), IMP (d), AMK (e), ATM (f) or CAZ (g) against strain j. (IV) CST was combined with TGC (a), VAN (b), RIF (c), IMP (d), AMK (e), ATM (f) or CAZ (g) against strain k. (V) CST was combined with TGC (a), VAN (b), RIF (c), IMP (d), AMK (e), ATM (f) or CAZ (g) against strain m.

(I)

(a)

| CST MIC Alone ( mg/ml) | TGC MIC Alone ( mg/ml) | CST MIC in combination ( mg/ml) | TGC MIC in combination ( mg/ml) | FIC_CST_ | FIC_TGC_ | FICI | FICI Mean |
| --- | --- | --- | --- | --- | --- | --- | --- |
| 1024 | 1 | 1024 | 0 | 1 | 0 | 1 | 1.79557292 |
| 1024 | 1 | 512 | 0.0625 | 0.5 | 0.0625 | 0.5625 |  |
| 1024 | 1 | 512 | 0.125 | 0.5 | 0.125 | 0.625 |  |
| 1024 | 1 | 1024 | 0.25 | 1 | 0.25 | 1.25 |  |
| 1024 | 1 | 2048 | 0.5 | 2 | 0.5 | 2.5 |  |
| 1024 | 1 | 1024 | 1 | 1 | 1 | 2 |  |
| 1024 | 1 | 512 | 2 | 0.5 | 2 | 2.5 |  |
| 1024 | 1 | 256 | 2 | 0.25 | 2 | 2.25 |  |
| 1024 | 1 | 128 | 2 | 0.125 | 2 | 2.125 |  |
| 1024 | 1 | 64 | 2 | 0.0625 | 2 | 2.0625 |  |
| 1024 | 1 | 32 | 2 | 0.03125 | 2 | 2.03125 |  |
| 1024 | 1 | 16 | 2 | 0.015625 | 2 | 2.015625 |  |
| 1024 | 1 | 8 | 2 | 0.0078125 | 2 | 2.0078125 |  |
| 1024 | 1 | 4 | 2 | 0.00390625 | 2 | 2.00390625 |  |
| 1024 | 1 | 0 | 2 | 0 | 2 | 2 |  |

(b)

| CST MIC Alone  (mg/ml) | VAN MIC Alone (mg/ml) | CST MIC in combination ( mg/ml) | VAN MIC in combination (mg/ml) | FIC_CST_ | FIC_VAN_ | FICI | FICI Mean |
| --- | --- | --- | --- | --- | --- | --- | --- |
| 1024 | 256 | 512 | 0 | 0.5 | 0 | 0.5 | 0.466086648 |
| 1024 | 256 | 128 | 16 | 0.125 | 0.0625 | 0.1875 |  |
| 1024 | 256 | 64 | 16 | 0.0625 | 0.0625 | 0.125 |  |
| 1024 | 256 | 32 | 32 | 0.03125 | 0.125 | 0.15625 |  |
| 1024 | 256 | 16 | 32 | 0.015625 | 0.125 | 0.140625 |  |
| 1024 | 256 | 8 | 32 | 0.0078125 | 0.125 | 0.1328125 |  |
| 1024 | 256 | 4 | 32 | 0.00390625 | 0.125 | 0.12890625 |  |
| 1024 | 256 | 2 | 64 | 0.00195313 | 0.25 | 0.25195313 |  |
| 1024 | 256 | 2 | 128 | 0.00195313 | 0.5 | 0.50195313 |  |
| 1024 | 256 | 2 | 256 | 0.00195313 | 1 | 1.00195313 |  |
| 1024 | 256 | 0 | 512 | 0 | 2 | 2 |  |

(c)

| CST MIC Alone  (mg/ml) | RIF MIC Alone (mg/ml) | CST MIC in combination ( mg/ml) | RIF MIC in combination (mg/ml) | FIC_CST_ | FIC_RIF_ | FICI | FICI Mean |
| --- | --- | --- | --- | --- | --- | --- | --- |
| 1024 | 2 | 512 | 0 | 0.5 | 0 | 0.5 | 0.329900568 |
| 1024 | 2 | 256 | 0.25 | 0.25 | 0.125 | 0.375 |  |
| 1024 | 2 | 128 | 0.25 | 0.125 | 0.125 | 0.25 |  |
| 1024 | 2 | 64 | 0.25 | 0.0625 | 0.125 | 0.1875 |  |
| 1024 | 2 | 32 | 0.25 | 0.03125 | 0.125 | 0.15625 |  |
| 1024 | 2 | 16 | 0.25 | 0.015625 | 0.125 | 0.140625 |  |
| 1024 | 2 | 8 | 0.25 | 0.0078125 | 0.125 | 0.1328125 |  |
| 1024 | 2 | 4 | 0.25 | 0.00390625 | 0.125 | 0.12890625 |  |
| 1024 | 2 | 4 | 0.5 | 0.00390625 | 0.25 | 0.25390625 |  |
| 1024 | 2 | 4 | 1 | 0.00390625 | 0.5 | 0.50390625 |  |
| 1024 | 2 | 0 | 2 | 0 | 1 | 1 |  |

(d)

| CST MIC Alone  (mg/ml) | IMP MIC Alone (mg/ml) | CST MIC in combination ( mg/ml) | IMP MIC in combination (mg/ml) | FIC_CST_ | FIC_IMP_ | FICI | FICI Mean |
| --- | --- | --- | --- | --- | --- | --- | --- |
| 1024 | 64 | 512 | 0 | 0.5 | 0 | 0.5 | 0.79296875 |
| 1024 | 64 | 512 | 4 | 0.5 | 0.0625 | 0.5625 |  |
| 1024 | 64 | 256 | 8 | 0.25 | 0.125 | 0.375 |  |
| 1024 | 64 | 256 | 16 | 0.25 | 0.25 | 0.5 |  |
| 1024 | 64 | 128 | 32 | 0.125 | 0.5 | 0.625 |  |
| 1024 | 64 | 64 | 64 | 0.0625 | 1 | 1.0625 |  |
| 1024 | 64 | 32 | 64 | 0.03125 | 1 | 1.03125 |  |
| 1024 | 64 | 16 | 64 | 0.015625 | 1 | 1.015625 |  |
| 1024 | 64 | 8 | 64 | 0.0078125 | 1 | 1.0078125 |  |
| 1024 | 64 | 4 | 64 | 0.00390625 | 1 | 1.00390625 |  |
| 1024 | 64 | 40 | 64 | 0.0390625 | 1 | 1.0390625 |  |

(e)

| CST MIC Alone  (mg/ml) | AMK MIC Alone (mg/ml) | CST MIC in combination ( mg/ml) | AMK MIC in combination (mg/ml) | FIC_CST_ | FIC_AMK_ | FICI | FICI Mean |
| --- | --- | --- | --- | --- | --- | --- | --- |
| 1024 | 4096 | 512 | 0 | 0.5 | 0 | 0.5 | 2.0984375 |
| 1024 | 4096 | 512 | 64 | 0.5 | 0.015625 | 0.515625 |  |
| 1024 | 4096 | 512 | 128 | 0.5 | 0.03125 | 0.53125 |  |
| 1024 | 4096 | 1024 | 256 | 1 | 0.0625 | 1.0625 |  |
| 1024 | 4096 | 1024 | 512 | 1 | 0.125 | 1.125 |  |
| 1024 | 4096 | 1024 | 1024 | 1 | 0.25 | 1.25 |  |
| 1024 | 4096 | 2048 | 1024 | 2 | 0.25 | 2.25 |  |
| 1024 | 4096 | 4096 | 1024 | 4 | 0.25 | 4.25 |  |
| 1024 | 4096 | 4096 | 2048 | 4 | 0.5 | 4.5 |  |
| 1024 | 4096 | 4096 | 4096 | 4 | 1 | 5 |  |

(f)

| CST MIC Alone  (mg/ml) | ATM MIC Alone (mg/ml) | CST MIC in combination ( mg/ml) | ATM MIC in combination (mg/ml) | FIC_CST_ | FIC_ATM_ | FICI | FICI Mean |
| --- | --- | --- | --- | --- | --- | --- | --- |
| 1024 | 128 | 512 | 0 | 0.5 | 0 | 0.5 | 0.367621528 |
| 1024 | 128 | 256 | 8 | 0.25 | 0.0625 | 0.3125 |  |
| 1024 | 128 | 128 | 8 | 0.125 | 0.0625 | 0.1875 |  |
| 1024 | 128 | 64 | 8 | 0.0625 | 0.0625 | 0.125 |  |
| 1024 | 128 | 32 | 16 | 0.03125 | 0.125 | 0.15625 |  |
| 1024 | 128 | 16 | 32 | 0.015625 | 0.25 | 0.265625 |  |
| 1024 | 128 | 8 | 32 | 0.0078125 | 0.25 | 0.2578125 |  |
| 1024 | 128 | 4 | 64 | 0.00390625 | 0.5 | 0.50390625 |  |
| 1024 | 128 | 0 | 128 | 0 | 1 | 1 |  |

(g)

| CST MIC Alone  (mg/ml) | CAZ MIC Alone (mg/ml) | CST MIC in combination ( mg/ml) | CAZ MIC in combination (mg/ml) | FIC_CST_ | FIC_CAZ_ | FICI | FICI Mean |
| --- | --- | --- | --- | --- | --- | --- | --- |
| 1024 | 512 | 512 | 0 | 0.5 | 0 | 0.5 | 0.375 |
| 1024 | 512 | 256 | 32 | 0.25 | 0.0625 | 0.3125 |  |
| 1024 | 512 | 128 | 32 | 0.125 | 0.0625 | 0.1875 |  |
| 1024 | 512 | 128 | 64 | 0.125 | 0.125 | 0.25 |  |
| 1024 | 512 | 64 | 128 | 0.0625 | 0.25 | 0.3125 |  |
| 1024 | 512 | 32 | 128 | 0.03125 | 0.25 | 0.28125 |  |
| 1024 | 512 | 16 | 128 | 0.015625 | 0.25 | 0.265625 |  |
| 1024 | 512 | 8 | 128 | 0.0078125 | 0.25 | 0.2578125 |  |
| 1024 | 512 | 4 | 128 | 0.00390625 | 0.25 | 0.25390625 |  |
| 1024 | 512 | 4 | 256 | 0.00390625 | 0.5 | 0.50390625 |  |
| 1024 | 512 | 0 | 512 | 0 | 1 | 1 |  |

(II)

(a)

| CST MIC Alone ( mg/ml) | TGC MIC Alone ( mg/ml) | CST MIC in combination ( mg/ml) | TGC MIC in combination ( mg/ml) | FIC_CST_ | FIC_TGC_ | FICI | FICI Mean |
| --- | --- | --- | --- | --- | --- | --- | --- |
| 1024 | 1 | 1024 | 0 | 1 | 0 | 1 | 1.43722098 |
| 1024 | 1 | 1024 | 0.00390625 | 1 | 0.00390625 | 1.00390625 |  |
| 1024 | 1 | 1024 | 0.0078125 | 1 | 0.0078125 | 1.0078125 |  |
| 1024 | 1 | 1024 | 0.015625 | 1 | 0.015625 | 1.015625 |  |
| 1024 | 1 | 1024 | 0.03125 | 1 | 0.03125 | 1.03125 |  |
| 1024 | 1 | 2048 | 0.0625 | 2 | 0.0625 | 2.0625 |  |
| 1024 | 1 | 2048 | 0.125 | 2 | 0.125 | 2.125 |  |
| 1024 | 1 | 4096 | 0.25 | 4 | 0.25 | 4.25 |  |
| 1024 | 1 | 2048 | 0.5 | 2 | 0.5 | 2.5 |  |
| 1024 | 1 | 1024 | 0.5 | 1 | 0.5 | 1.5 |  |
| 1024 | 1 | 512 | 0.5 | 0.5 | 0.5 | 1 |  |
| 1024 | 1 | 256 | 0.5 | 0.25 | 0.5 | 0.75 |  |
| 1024 | 1 | 128 | 0.5 | 0.125 | 0.5 | 0.625 |  |
| 1024 | 1 | 0 | 0.25 | 0 | 0.25 | 0.25 |  |

(b)

| CST MIC Alone  (mg/ml) | VAN MIC Alone (mg/ml) | CST MIC in combination ( mg/ml) | VAN MIC in combination (mg/ml) | FIC_CST_ | FIC_VAN_ | FICI | FICI Mean |
| --- | --- | --- | --- | --- | --- | --- | --- |
| 1024 | 256 | 1024 | 0 | 1 | 0 | 1 | 0.421164773 |
| 1024 | 256 | 512 | 32 | 0.5 | 0.125 | 0.625 |  |
| 1024 | 256 | 256 | 32 | 0.25 | 0.125 | 0.375 |  |
| 1024 | 256 | 128 | 32 | 0.125 | 0.125 | 0.25 |  |
| 1024 | 256 | 64 | 32 | 0.0625 | 0.125 | 0.1875 |  |
| 1024 | 256 | 32 | 32 | 0.03125 | 0.125 | 0.15625 |  |
| 1024 | 256 | 16 | 32 | 0.015625 | 0.125 | 0.140625 |  |
| 1024 | 256 | 8 | 32 | 0.0078125 | 0.125 | 0.1328125 |  |
| 1024 | 256 | 8 | 64 | 0.0078125 | 0.25 | 0.2578125 |  |
| 1024 | 256 | 8 | 128 | 0.0078125 | 0.5 | 0.5078125 |  |
| 1024 | 256 | 0 | 256 | 0 | 1 | 1 |  |

(c)

| CST MIC Alone  (mg/ml) | RIF MIC Alone (mg/ml) | CST MIC in combination ( mg/ml) | RIF MIC in combination (mg/ml) | FIC_CST_ | FIC_RIF_ | FICI | FICI Mean |
| --- | --- | --- | --- | --- | --- | --- | --- |
| 1024 | 4 | 1024 | 0 | 1 | 0 | 1 | 0.39615885 |
| 1024 | 4 | 512 | 0.015625 | 0.5 | 0.00390625 | 0.50390625 |  |
| 1024 | 4 | 256 | 0.015625 | 0.25 | 0.00390625 | 0.25390625 |  |
| 1024 | 4 | 128 | 0.015625 | 0.125 | 0.00390625 | 0.12890625 |  |
| 1024 | 4 | 128 | 0.03125 | 0.125 | 0.0078125 | 0.1328125 |  |
| 1024 | 4 | 128 | 0.0625 | 0.125 | 0.015625 | 0.140625 |  |
| 1024 | 4 | 128 | 0.125 | 0.125 | 0.03125 | 0.15625 |  |
| 1024 | 4 | 128 | 0.25 | 0.125 | 0.0625 | 0.1875 |  |
| 1024 | 4 | 128 | 0.5 | 0.125 | 0.125 | 0.25 |  |
| 1024 | 4 | 128 | 1 | 0.125 | 0.25 | 0.375 |  |
| 1024 | 4 | 128 | 2 | 0.125 | 0.5 | 0.625 |  |
| 1024 | 4 | 0 | 4 | 0 | 1 | 1 |  |

(d)

| CST MIC Alone  (mg/ml) | IMP MIC Alone (mg/ml) | CST MIC in combination ( mg/ml) | IMP MIC in combination (mg/ml) | FIC_CST_ | FIC_IMP_ | FICI | FICI Mean |
| --- | --- | --- | --- | --- | --- | --- | --- |
| 1024 | 64 | 512 | 0 | 0.5 | 0 | 0.5 | 0.37252103 |
| 1024 | 64 | 512 | 0.0625 | 0.5 | 0.00097656 | 0.50097656 |  |
| 1024 | 64 | 512 | 0.125 | 0.5 | 0.00195313 | 0.50195313 |  |
| 1024 | 64 | 256 | 0.25 | 0.25 | 0.00390625 | 0.25390625 |  |
| 1024 | 64 | 256 | 0.5 | 0.25 | 0.0078125 | 0.2578125 |  |
| 1024 | 64 | 256 | 1 | 0.25 | 0.015625 | 0.265625 |  |
| 1024 | 64 | 256 | 2 | 0.25 | 0.03125 | 0.28125 |  |
| 1024 | 64 | 128 | 4 | 0.125 | 0.0625 | 0.1875 |  |
| 1024 | 64 | 64 | 4 | 0.0625 | 0.0625 | 0.125 |  |
| 1024 | 64 | 32 | 8 | 0.03125 | 0.125 | 0.15625 |  |
| 1024 | 64 | 32 | 16 | 0.03125 | 0.25 | 0.28125 |  |
| 1024 | 64 | 32 | 32 | 0.03125 | 0.5 | 0.53125 |  |
| 1024 | 64 | 0 | 64 | 0 | 1 | 1 |  |

(e)

| CST MIC Alone  (mg/ml) | AMK MIC Alone (mg/ml) | CST MIC in combination ( mg/ml) | AMK MIC in combination (mg/ml) | FIC_CST_ | FIC_AMK_ | FICI | FICI Mean |
| --- | --- | --- | --- | --- | --- | --- | --- |
| 1024 | 4096 | 512 | 0 | 0.5 | 0 | 0.5 | 1.09082031 |
| 1024 | 4096 | 512 | 4 | 0.5 | 0.00097656 | 0.50097656 |  |
| 1024 | 4096 | 512 | 8 | 0.5 | 0.00195313 | 0.50195313 |  |
| 1024 | 4096 | 512 | 16 | 0.5 | 0.00390625 | 0.50390625 |  |
| 1024 | 4096 | 512 | 32 | 0.5 | 0.0078125 | 0.5078125 |  |
| 1024 | 4096 | 512 | 64 | 0.5 | 0.015625 | 0.515625 |  |
| 1024 | 4096 | 512 | 128 | 0.5 | 0.03125 | 0.53125 |  |
| 1024 | 4096 | 512 | 256 | 0.5 | 0.0625 | 0.5625 |  |
| 1024 | 4096 | 1024 | 512 | 1 | 0.125 | 1.125 |  |
| 1024 | 4096 | 2048 | 1024 | 2 | 0.25 | 2.25 |  |
| 1024 | 4096 | 4096 | 2048 | 4 | 0.5 | 4.5 |  |

(f)

| CST MIC Alone  (mg/ml) | ATM MIC Alone (mg/ml) | CST MIC in combination ( mg/ml) | ATM MIC in combination (mg/ml) | FIC_CST_ | FIC_ATM_ | FICI | FICI Mean |
| --- | --- | --- | --- | --- | --- | --- | --- |
| 1024 | 256 | 128 | 0 | 0.125 | 0 | 0.125 | 0.481770833 |
| 1024 | 256 | 64 | 8 | 0.0625 | 0.03125 | 0.09375 |  |
| 1024 | 256 | 32 | 8 | 0.03125 | 0.03125 | 0.0625 |  |
| 1024 | 256 | 32 | 16 | 0.03125 | 0.0625 | 0.09375 |  |
| 1024 | 256 | 32 | 32 | 0.03125 | 0.125 | 0.15625 |  |
| 1024 | 256 | 32 | 64 | 0.03125 | 0.25 | 0.28125 |  |
| 1024 | 256 | 16 | 128 | 0.015625 | 0.5 | 0.515625 |  |
| 1024 | 256 | 8 | 256 | 0.0078125 | 1 | 1.0078125 |  |
| 1024 | 256 | 0 | 512 | 0 | 2 | 2 |  |

(g)

| CST MIC Alone  (mg/ml) | CAZ MIC Alone (mg/ml) | CST MIC in combination ( mg/ml) | CAZ MIC in combination (mg/ml) | FIC_CST_ | FIC_CAZ_ | FICI | FICI Mean |
| --- | --- | --- | --- | --- | --- | --- | --- |
| 1024 | 512 | 512 | 0 | 0.5 | 0 | 0.5 | 0.280273438 |
| 1024 | 512 | 256 | 32 | 0.25 | 0.0625 | 0.3125 |  |
| 1024 | 512 | 128 | 32 | 0.125 | 0.0625 | 0.1875 |  |
| 1024 | 512 | 64 | 64 | 0.0625 | 0.125 | 0.1875 |  |
| 1024 | 512 | 32 | 64 | 0.03125 | 0.125 | 0.15625 |  |
| 1024 | 512 | 16 | 64 | 0.015625 | 0.125 | 0.140625 |  |
| 1024 | 512 | 8 | 128 | 0.0078125 | 0.25 | 0.2578125 |  |
| 1024 | 512 | 0 | 256 | 0 | 0.5 | 0.5 |  |

**(III)**

(a)

| CST MIC Alone ( mg/ml) | TGC MIC Alone ( mg/ml) | CST MIC in combination ( mg/ml) | TGC MIC in combination ( mg/ml) | FIC_CST_ | FIC_TGC_ | FICI | FICI Mean |
| --- | --- | --- | --- | --- | --- | --- | --- |
| 1024 | 4 | 1024 | 0 | 1 | 0 | 1 | 0.97460938 |
| 1024 | 4 | 512 | 0.0625 | 0.5 | 0.015625 | 0.515625 |  |
| 1024 | 4 | 1024 | 0.125 | 1 | 0.03125 | 1.03125 |  |
| 1024 | 4 | 2048 | 0.25 | 2 | 0.0625 | 2.0625 |  |
| 1024 | 4 | 1024 | 0.5 | 1 | 0.125 | 1.125 |  |
| 1024 | 4 | 128 | 1 | 0.125 | 0.25 | 0.375 |  |
| 1024 | 4 | 128 | 2 | 0.125 | 0.5 | 0.625 |  |
| 1024 | 4 | 64 | 4 | 0.0625 | 1 | 1.0625 |  |

(b)

| CST MIC Alone  (mg/ml) | VAN MIC Alone (mg/ml) | CST MIC in combination ( mg/ml) | VAN MIC in combination (mg/ml) | FIC_CST_ | FIC_VAN_ | FICI | FICI Mean |
| --- | --- | --- | --- | --- | --- | --- | --- |
| 1024 | 2048 | 512 | 0 | 0.5 | 0 | 0.5 | 0.36523438 |
| 1024 | 2048 | 64 | 32 | 0.0625 | 0.015625 | 0.078125 |  |
| 1024 | 2048 | 64 | 64 | 0.0625 | 0.03125 | 0.09375 |  |
| 1024 | 2048 | 64 | 128 | 0.0625 | 0.0625 | 0.125 |  |
| 1024 | 2048 | 64 | 256 | 0.0625 | 0.125 | 0.1875 |  |
| 1024 | 2048 | 64 | 512 | 0.0625 | 0.25 | 0.3125 |  |
| 1024 | 2048 | 64 | 1024 | 0.0625 | 0.5 | 0.5625 |  |
| 1024 | 2048 | 64 | 2048 | 0.0625 | 1 | 1.0625 |  |

(c)

| CST MIC Alone  (mg/ml) | RIF MIC Alone (mg/ml) | CST MIC in combination ( mg/ml) | RIF MIC in combination (mg/ml) | FIC_CST_ | FIC_RIF_ | FICI | FICI Mean |
| --- | --- | --- | --- | --- | --- | --- | --- |
| 512 | 1 | 512 | 0 | 1 | 0 | 1 | 1.06818182 |
| 512 | 1 | 512 | 0.0625 | 1 | 0.0625 | 1.0625 |  |
| 512 | 1 | 256 | 0.0625 | 0.5 | 0.0625 | 0.5625 |  |
| 512 | 1 | 128 | 0.0625 | 0.25 | 0.0625 | 0.3125 |  |
| 512 | 1 | 64 | 0.0625 | 0.125 | 0.0625 | 0.1875 |  |
| 512 | 1 | 64 | 0.125 | 0.125 | 0.125 | 0.25 |  |
| 512 | 1 | 64 | 0.25 | 0.125 | 0.25 | 0.375 |  |
| 512 | 1 | 64 | 0.5 | 0.125 | 0.5 | 0.625 |  |
| 512 | 1 | 64 | 1 | 0.125 | 1 | 1.125 |  |
| 512 | 1 | 64 | 2 | 0.125 | 2 | 2.125 |  |
| 512 | 1 | 64 | 4 | 0.125 | 4 | 4.125 |  |

(d)

| CST MIC Alone  (mg/ml) | IMP MIC Alone (mg/ml) | CST MIC in combination ( mg/ml) | IMP MIC in combination (mg/ml) | FIC_CST_ | FIC_IMP_ | FICI | FICI Mean |
| --- | --- | --- | --- | --- | --- | --- | --- |
| 1024 | 64 | 1024 | 0 | 1 | 0 | 1 | 0.598958333 |
| 1024 | 64 | 1024 | 1 | 1 | 0.015625 | 1.015625 |  |
| 1024 | 64 | 512 | 2 | 0.5 | 0.03125 | 0.53125 |  |
| 1024 | 64 | 256 | 2 | 0.25 | 0.03125 | 0.28125 |  |
| 1024 | 64 | 128 | 4 | 0.125 | 0.0625 | 0.1875 |  |
| 1024 | 64 | 128 | 8 | 0.125 | 0.125 | 0.25 |  |
| 1024 | 64 | 128 | 16 | 0.125 | 0.25 | 0.375 |  |
| 1024 | 64 | 128 | 32 | 0.125 | 0.5 | 0.625 |  |
| 1024 | 64 | 128 | 64 | 0.125 | 1 | 1.125 |  |

(e)

| CST MIC Alone  (mg/ml) | AMK MIC Alone (mg/ml) | CST MIC in combination ( mg/ml) | AMK MIC in combination (mg/ml) | FIC_CST_ | FIC_AMK_ | FICI | FICI Mean |
| --- | --- | --- | --- | --- | --- | --- | --- |
| 1024 | 4098 | 1024 | 0 | 1 | 0 | 1 | 1.74792582 |
| 1024 | 4098 | 1024 | 64 | 1 | 0.01561737 | 1.01561737 |  |
| 1024 | 4098 | 1024 | 128 | 1 | 0.03123475 | 1.03123475 |  |
| 1024 | 4098 | 1024 | 256 | 1 | 0.0624695 | 1.0624695 |  |
| 1024 | 4098 | 1024 | 512 | 1 | 0.12493899 | 1.12493899 |  |
| 1024 | 4098 | 2048 | 1024 | 1 | 0.24987799 | 1.24987799 |  |
| 1024 | 4098 | 2048 | 2048 | 2 | 0.49975598 | 2.49975598 |  |
| 1024 | 4098 | 4096 | 4096 | 4 | 0.99951196 | 4.99951196 |  |

(f)

| CST MIC Alone  (mg/ml) | ATM MIC Alone (mg/ml) | CST MIC in combination ( mg/ml) | ATM MIC in combination (mg/ml) | FIC_CST_ | FIC_ATM_ | FICI | FICI Mean |
| --- | --- | --- | --- | --- | --- | --- | --- |
| 512 | 64 | 512 | 0 | 1 | 0 | 1 | 0.5984375 |
| 512 | 64 | 256 | 4 | 0.5 | 0.0625 | 0.5625 |  |
| 512 | 64 | 128 | 4 | 0.25 | 0.0625 | 0.3125 |  |
| 512 | 64 | 128 | 8 | 0.25 | 0.125 | 0.375 |  |
| 512 | 64 | 128 | 16 | 0.25 | 0.25 | 0.5 |  |
| 512 | 64 | 64 | 32 | 0.125 | 0.5 | 0.625 |  |
| 512 | 64 | 32 | 32 | 0.0625 | 0.5 | 0.5625 |  |
| 512 | 64 | 16 | 32 | 0.03125 | 0.5 | 0.53125 |  |
| 512 | 64 | 8 | 32 | 0.015625 | 0.5 | 0.515625 |  |
| 512 | 64 | 0 | 64 | 0 | 1 | 1 |  |

(g)

| CST MIC Alone  (mg/ml) | CAZ MIC Alone (mg/ml) | CST MIC in combination ( mg/ml) | CAZ MIC in combination (mg/ml) | FIC_CST_ | FIC_CAZ_ | FICI | FICI Mean |
| --- | --- | --- | --- | --- | --- | --- | --- |
| 1024 | 256 | 1024 | 0 | 1 | 0 | 1 | 0.45 |
| 1024 | 256 | 512 | 32 | 0.5 | 0.125 | 0.625 |  |
| 1024 | 256 | 256 | 32 | 0.25 | 0.125 | 0.375 |  |
| 1024 | 256 | 128 | 32 | 0.125 | 0.125 | 0.25 |  |
| 1024 | 256 | 64 | 32 | 0.0625 | 0.125 | 0.1875 |  |
| 1024 | 256 | 32 | 32 | 0.03125 | 0.125 | 0.15625 |  |
| 1024 | 256 | 16 | 32 | 0.015625 | 0.125 | 0.140625 |  |
| 1024 | 256 | 8 | 64 | 0.0078125 | 0.25 | 0.2578125 |  |
| 1024 | 256 | 8 | 128 | 0.0078125 | 0.5 | 0.5078125 |  |
| 1024 | 256 | 0 | 256 | 0 | 1 | 1 |  |

(IV)

| CST MIC Alone  (mg/ml) | TGC MIC Alone ( mg/ml) | CST MIC in combination ( mg/ml) | TGC MIC in combination ( mg/ml) | FIC_CST_ | FIC_TGC_ | FICI | FICI Mean |
| --- | --- | --- | --- | --- | --- | --- | --- |
| 1024 | 2 | 1024 | 0 | 1 | 0 | 1 | 1.99560547 |
| 1024 | 2 | 1024 | 0.125 | 1 | 0.0625 | 1.0625 |  |
| 1024 | 2 | 1024 | 0.25 | 1 | 0.125 | 1.125 |  |
| 1024 | 2 | 1024 | 0.5 | 1 | 0.25 | 1.25 |  |
| 1024 | 2 | 2048 | 1 | 2 | 0.5 | 2.5 |  |
| 1024 | 2 | 2048 | 2 | 2 | 1 | 3 |  |
| 1024 | 2 | 2048 | 4 | 2 | 2 | 4 |  |
| 1024 | 2 | 1024 | 4 | 1 | 2 | 3 |  |
| 1024 | 2 | 512 | 4 | 0.5 | 2 | 2.5 |  |
| 1024 | 2 | 256 | 4 | 0.25 | 2 | 2.25 |  |
| 1024 | 2 | 128 | 4 | 0.125 | 2 | 2.125 |  |
| 1024 | 2 | 64 | 4 | 0.0625 | 2 | 2.0625 |  |
| 1024 | 2 | 32 | 4 | 0.03125 | 2 | 2.03125 |  |
| 1024 | 2 | 16 | 4 | 0.015625 | 2 | 2.015625 |  |
| 1024 | 2 | 8 | 2 | 0.0078125 | 1 | 1.0078125 |  |
| 1024 | 2 | 0 | 2 | 0 | 1 | 1 |  |

(a)


(b)

| CST MIC Alone  (mg/ml) | VAN MIC Alone (mg/ml) | CST MIC in combination ( mg/ml) | VAN MIC in combination (mg/ml) | FIC_CST_ | FIC_VAN_ | FICI | FICI Mean |
| --- | --- | --- | --- | --- | --- | --- | --- |
| 1024 | 512 | 1024 | 0 | 1 | 0 | 1 | 0.36067708 |
| 1024 | 512 | 512 | 32 | 0.5 | 0.0625 | 0.5625 |  |
| 1024 | 512 | 256 | 32 | 0.25 | 0.0625 | 0.3125 |  |
| 1024 | 512 | 128 | 32 | 0.125 | 0.0625 | 0.1875 |  |
| 1024 | 512 | 64 | 32 | 0.0625 | 0.0625 | 0.125 |  |
| 1024 | 512 | 32 | 32 | 0.03125 | 0.0625 | 0.09375 |  |
| 1024 | 512 | 16 | 32 | 0.015625 | 0.0625 | 0.078125 |  |
| 1024 | 512 | 8 | 32 | 0.0078125 | 0.0625 | 0.0703125 |  |
| 1024 | 512 | 8 | 64 | 0.0078125 | 0.125 | 0.1328125 |  |
| 1024 | 512 | 8 | 128 | 0.0078125 | 0.25 | 0.2578125 |  |
| 1024 | 512 | 8 | 256 | 0.0078125 | 0.5 | 0.5078125 |  |
| 1024 | 512 | 0 | 512 | 0 | 1 | 1 |  |


(c)

| CST MIC Alone  (mg/ml) | RIF MIC Alone (mg/ml) | CST MIC in combination ( mg/ml) | RIF MIC in combination (mg/ml) | FIC_CST_ | FIC_RIF_ | FICI | FICI Mean |
| --- | --- | --- | --- | --- | --- | --- | --- |
| 1024 | 16 | 1024 | 0 | 1 | 0 | 1 | 0.47005208 |
| 1024 | 16 | 512 | 2 | 0.5 | 0.125 | 0.625 |  |
| 1024 | 16 | 256 | 2 | 0.25 | 0.125 | 0.375 |  |
| 1024 | 16 | 128 | 2 | 0.125 | 0.125 | 0.25 |  |
| 1024 | 16 | 64 | 2 | 0.0625 | 0.125 | 0.1875 |  |
| 1024 | 16 | 32 | 2 | 0.03125 | 0.125 | 0.15625 |  |
| 1024 | 16 | 16 | 2 | 0.015625 | 0.125 | 0.140625 |  |
| 1024 | 16 | 8 | 2 | 0.0078125 | 0.125 | 0.1328125 |  |
| 1024 | 16 | 8 | 4 | 0.0078125 | 0.25 | 0.2578125 |  |
| 1024 | 16 | 8 | 8 | 0.0078125 | 0.5 | 0.5078125 |  |
| 1024 | 16 | 8 | 16 | 0.0078125 | 1 | 1.0078125 |  |
| 1024 | 16 | 0 | 16 | 0 | 1 | 1 |  |

(d)

| CST MIC Alone  (mg/ml) | IMP MIC Alone (mg/ml) | CST MIC in combination ( mg/ml) | IMP MIC in combination (mg/ml) | FIC_CST_ | FIC_IMP_ | FICI | FICI Mean |
| --- | --- | --- | --- | --- | --- | --- | --- |
| 256 | 32 | 256 | 0 | 1 | 0 | 1 | 0.576388889 |
| 256 | 32 | 128 | 2 | 0.5 | 0.0625 | 0.5625 |  |
| 256 | 32 | 128 | 4 | 0.5 | 0.125 | 0.625 |  |
| 256 | 32 | 64 | 8 | 0.25 | 0.25 | 0.5 |  |
| 256 | 32 | 32 | 8 | 0.125 | 0.25 | 0.375 |  |
| 256 | 32 | 16 | 8 | 0.0625 | 0.25 | 0.3125 |  |
| 256 | 32 | 8 | 8 | 0.03125 | 0.25 | 0.28125 |  |
| 256 | 32 | 8 | 16 | 0.03125 | 0.5 | 0.53125 |  |
| 256 | 32 | 0 | 32 | 0 | 1 | 1 |  |

(e)

| CST MIC Alone  (mg/ml) | AMK MIC Alone (mg/ml) | CST MIC in combination ( mg/ml) | AMK MIC in combination (mg/ml) | FIC_CST_ | FIC_AMK_ | FICI | FICI Mean |
| --- | --- | --- | --- | --- | --- | --- | --- |
| 258 | 4096 | 256 | 256 | 0.99224806 | 0.0625 | 1.05474806 | 4.336186477 |
| 258 | 4096 | 256 | 256 | 0.99224806 | 0.0625 | 1.05474806 |  |
| 258 | 4096 | 256 | 256 | 0.99224806 | 0.0625 | 1.05474806 |  |
| 258 | 4096 | 512 | 512 | 1.98449612 | 0.125 | 2.10949612 |  |
| 258 | 4096 | 512 | 512 | 1.98449612 | 0.125 | 2.10949612 |  |
| 258 | 4096 | 512 | 512 | 1.98449612 | 0.125 | 2.10949612 |  |
| 258 | 4096 | 1024 | 1024 | 3.96899225 | 0.25 | 4.21899225 |  |
| 258 | 4096 | 2048 | 2048 | 7.9379845 | 0.5 | 8.4379845 |  |
| 258 | 4096 | 4096 | 4096 | 15.875969 | 1 | 16.875969 |  |

(f)

| CST MIC Alone  (mg/ml) | ATM MIC Alone (mg/ml) | CST MIC in combination ( mg/ml) | ATM MIC in combination (mg/ml) | FIC_CST_ | FIC_ATM_ | FICI | FICI Mean |
| --- | --- | --- | --- | --- | --- | --- | --- |
| 1024 | 256 | 1024 | 0 | 1 | 0 | 1 | 0.36067708 |
| 1024 | 256 | 512 | 16 | 0.5 | 0.0625 | 0.5625 |  |
| 1024 | 256 | 256 | 16 | 0.25 | 0.0625 | 0.3125 |  |
| 1024 | 256 | 128 | 16 | 0.125 | 0.0625 | 0.1875 |  |
| 1024 | 256 | 64 | 16 | 0.0625 | 0.0625 | 0.125 |  |
| 1024 | 256 | 32 | 16 | 0.03125 | 0.0625 | 0.09375 |  |
| 1024 | 256 | 16 | 16 | 0.015625 | 0.0625 | 0.078125 |  |
| 1024 | 256 | 8 | 16 | 0.0078125 | 0.0625 | 0.0703125 |  |
| 1024 | 256 | 8 | 32 | 0.0078125 | 0.125 | 0.1328125 |  |
| 1024 | 256 | 8 | 64 | 0.0078125 | 0.25 | 0.2578125 |  |
| 1024 | 256 | 8 | 128 | 0.0078125 | 0.5 | 0.5078125 |  |
| 1024 | 256 | 0 | 256 | 0 | 1 | 1 |  |

(g)

| CST MIC Alone  (mg/ml) | CAZ MIC Alone (mg/ml) | CST MIC in combination ( mg/ml) | CAZ MIC in combination (mg/ml) | FIC_CST_ | FIC_CAZ_ | FICI | FICI Mean |
| --- | --- | --- | --- | --- | --- | --- | --- |
| 1024 | 512 | 1024 | 0 | 1 | 0 | 1 | 0.37630208 |
| 1024 | 512 | 512 | 32 | 0.5 | 0.0625 | 0.5625 |  |
| 1024 | 512 | 256 | 32 | 0.25 | 0.0625 | 0.3125 |  |
| 1024 | 512 | 128 | 32 | 0.125 | 0.0625 | 0.1875 |  |
| 1024 | 512 | 64 | 32 | 0.0625 | 0.0625 | 0.125 |  |
| 1024 | 512 | 32 | 64 | 0.03125 | 0.125 | 0.15625 |  |
| 1024 | 512 | 16 | 64 | 0.015625 | 0.125 | 0.140625 |  |
| 1024 | 512 | 8 | 64 | 0.0078125 | 0.125 | 0.1328125 |  |
| 1024 | 512 | 8 | 64 | 0.0078125 | 0.125 | 0.1328125 |  |
| 1024 | 512 | 8 | 128 | 0.0078125 | 0.25 | 0.2578125 |  |
| 1024 | 512 | 8 | 256 | 0.0078125 | 0.5 | 0.5078125 |  |
| 1024 | 512 | 0 | 512 | 0 | 1 | 1 |  |

**(V)**

(a)

| CST MIC Alone ( mg/ml) | TGC MIC Alone ( mg/ml) | CST MIC in combination ( mg/ml) | TGC MIC in combination ( mg/ml) | FIC_CST_ | FIC_TGC_ | FICI | FICI Mean |
| --- | --- | --- | --- | --- | --- | --- | --- |
| 1024 | 1 | 1024 | 0 | 1 | 0 | 1 | 1.56668527 |
| 1024 | 1 | 1024 | 0.00390625 | 1 | 0.00390625 | 1.00390625 |  |
| 1024 | 1 | 1024 | 0.0078125 | 1 | 0.0078125 | 1.0078125 |  |
| 1024 | 1 | 1024 | 0.015625 | 1 | 0.015625 | 1.015625 |  |
| 1024 | 1 | 1024 | 0.03125 | 1 | 0.03125 | 1.03125 |  |
| 1024 | 1 | 2048 | 0.0625 | 2 | 0.0625 | 2.0625 |  |
| 1024 | 1 | 4096 | 0.125 | 4 | 0.125 | 4.125 |  |
| 1024 | 1 | 4096 | 0.25 | 4 | 0.25 | 4.25 |  |
| 1024 | 1 | 2048 | 0.0625 | 2 | 0.0625 | 2.0625 |  |
| 1024 | 1 | 1024 | 0.5 | 1 | 0.5 | 1.5 |  |
| 1024 | 1 | 512 | 0.5 | 0.5 | 0.5 | 1 |  |
| 1024 | 1 | 256 | 0.5 | 0.25 | 0.5 | 0.75 |  |
| 1024 | 1 | 128 | 0.5 | 0.125 | 0.5 | 0.625 |  |
| 1024 | 1 | 0 | 0.5 | 0 | 0.5 | 0.5 |  |


(b)

| CST MIC Alone  (mg/ml) | VAN MIC Alone (mg/ml) | CST MIC in combination ( mg/ml) | VAN MIC in combination (mg/ml) | FIC_CST_ | FIC_VAN_ | FICI | FICI Mean |
| --- | --- | --- | --- | --- | --- | --- | --- |
| 1024 | 256 | 1024 | 0 | 1 | 0 | 1 | 0.45 |
| 1024 | 256 | 512 | 32 | 0.5 | 0.125 | 0.625 |  |
| 1024 | 256 | 256 | 32 | 0.25 | 0.125 | 0.375 |  |
| 1024 | 256 | 128 | 32 | 0.125 | 0.125 | 0.25 |  |
| 1024 | 256 | 64 | 32 | 0.0625 | 0.125 | 0.1875 |  |
| 1024 | 256 | 32 | 32 | 0.03125 | 0.125 | 0.15625 |  |
| 1024 | 256 | 16 | 32 | 0.015625 | 0.125 | 0.140625 |  |
| 1024 | 256 | 8 | 64 | 0.0078125 | 0.25 | 0.2578125 |  |
| 1024 | 256 | 8 | 128 | 0.0078125 | 0.5 | 0.5078125 |  |
| 1024 | 256 | 0 | 256 | 0 | 1 | 1 |  |

(c)

| CST MIC Alone  (mg/ml) | RIF MIC Alone (mg/ml) | CST MIC in combination ( mg/ml) | RIF MIC in combination (mg/ml) | FIC_CST_ | FIC_RIF_ | FICI | FICI Mean |
| --- | --- | --- | --- | --- | --- | --- | --- |
| 1024 | 2 | 1024 | 0 | 1 | 0 | 1 | 0.42122159 |
| 1024 | 2 | 512 | 0.015625 | 0.5 | 0.0078125 | 0.5078125 |  |
| 1024 | 2 | 256 | 0.015625 | 0.25 | 0.0078125 | 0.2578125 |  |
| 1024 | 2 | 128 | 0.015625 | 0.125 | 0.0078125 | 0.1328125 |  |
| 1024 | 2 | 128 | 0.0325 | 0.125 | 0.01625 | 0.14125 |  |
| 1024 | 2 | 128 | 0.0625 | 0.125 | 0.03125 | 0.15625 |  |
| 1024 | 2 | 128 | 0.125 | 0.125 | 0.0625 | 0.1875 |  |
| 1024 | 2 | 128 | 0.25 | 0.125 | 0.125 | 0.25 |  |
| 1024 | 2 | 128 | 0.5 | 0.125 | 0.25 | 0.375 |  |
| 1024 | 2 | 128 | 1 | 0.125 | 0.5 | 0.625 |  |
| 1024 | 2 | 0 | 2 | 0 | 1 | 1 |  |

(d)

| CST MIC Alone  (mg/ml) | IMP MIC Alone (mg/ml) | CST MIC in combination ( mg/ml) | IMP MIC in combination (mg/ml) | FIC_CST_ | FIC_IMP_ | FICI | FICI Mean |
| --- | --- | --- | --- | --- | --- | --- | --- |
| 1024 | 64 | 1024 | 0 | 1 | 0 | 1 | 0.5546875 |
| 1024 | 64 | 512 | 1 | 0.5 | 0.015625 | 0.515625 |  |
| 1024 | 64 | 512 | 2 | 0.5 | 0.03125 | 0.53125 |  |
| 1024 | 64 | 512 | 4 | 0.5 | 0.0625 | 0.5625 |  |
| 1024 | 64 | 256 | 8 | 0.25 | 0.125 | 0.375 |  |
| 1024 | 64 | 128 | 8 | 0.125 | 0.125 | 0.25 |  |
| 1024 | 64 | 64 | 16 | 0.0625 | 0.25 | 0.3125 |  |
| 1024 | 64 | 32 | 32 | 0.03125 | 0.5 | 0.53125 |  |
| 1024 | 64 | 16 | 32 | 0.015625 | 0.5 | 0.515625 |  |
| 1024 | 64 | 8 | 32 | 0.0078125 | 0.5 | 0.5078125 |  |
| 1024 | 64 | 0 | 64 | 0 | 1 | 1 |  |

(e)

| CST MIC Alone  (mg/ml) | AMK MIC Alone (mg/ml) | CST MIC in combination ( mg/ml) | AMK MIC in combination (mg/ml) | FIC_CST_ | FIC_AMK_ | FICI | FICI Mean |
| --- | --- | --- | --- | --- | --- | --- | --- |
| 1024 | 4096 | 1024 | 0 | 1 | 0 | 1 | 1.63627486 |
| 1024 | 4096 | 1024 | 4 | 1 | 0.00097656 | 1.00097656 |  |
| 1024 | 4096 | 1024 | 8 | 1 | 0.00195313 | 1.00195313 |  |
| 1024 | 4096 | 1024 | 16 | 1 | 0.00390625 | 1.00390625 |  |
| 1024 | 4096 | 1024 | 32 | 1 | 0.0078125 | 1.0078125 |  |
| 1024 | 4096 | 1024 | 64 | 1 | 0.015625 | 1.015625 |  |
| 1024 | 4096 | 1024 | 128 | 1 | 0.03125 | 1.03125 |  |
| 1024 | 4096 | 2048 | 256 | 2 | 0.0625 | 2.0625 |  |
| 1024 | 4096 | 2048 | 512 | 2 | 0.125 | 2.125 |  |
| 1024 | 4096 | 2048 | 1024 | 2 | 0.25 | 2.25 |  |
| 1024 | 4096 | 4096 | 2048 | 4 | 0.5 | 4.5 |  |

(f)

| CST MIC Alone  (mg/ml) | ATM MIC Alone (mg/ml) | CST MIC in combination ( mg/ml) | ATM MIC in combination (mg/ml) | FIC_CST_ | FIC_ATM_ | FICI | FICI Mean |
| --- | --- | --- | --- | --- | --- | --- | --- |
| 1024 | 256 | 1024 | 0 | 1 | 0 | 1 | 0.503551136 |
| 1024 | 256 | 512 | 8 | 0.5 | 0.03125 | 0.53125 |  |
| 1024 | 256 | 256 | 16 | 0.25 | 0.0625 | 0.3125 |  |
| 1024 | 256 | 128 | 16 | 0.125 | 0.0625 | 0.1875 |  |
| 1024 | 256 | 64 | 32 | 0.0625 | 0.125 | 0.1875 |  |
| 1024 | 256 | 32 | 64 | 0.03125 | 0.25 | 0.28125 |  |
| 1024 | 256 | 16 | 64 | 0.015625 | 0.25 | 0.265625 |  |
| 1024 | 256 | 8 | 64 | 0.0078125 | 0.25 | 0.2578125 |  |
| 1024 | 256 | 8 | 128 | 0.0078125 | 0.5 | 0.5078125 |  |
| 1024 | 256 | 8 | 256 | 0.0078125 | 1 | 1.0078125 |  |
| 1024 | 256 | 0 | 256 | 0 | 1 | 1 |  |

(g)

| CST MIC Alone  (mg/ml) | CAZ MIC Alone (mg/ml) | CST MIC in combination ( mg/ml) | CAZ MIC in combination (mg/ml) | FIC_CST_ | FIC_CAZ_ | FICI | FICI Mean |
| --- | --- | --- | --- | --- | --- | --- | --- |
| 1024 | 512 | 1024 | 0 | 1 | 0 | 1 | 0.525 |
| 1024 | 512 | 512 | 32 | 0.5 | 0.0625 | 0.5625 |  |
| 1024 | 512 | 256 | 32 | 0.25 | 0.0625 | 0.3125 |  |
| 1024 | 512 | 128 | 64 | 0.125 | 0.125 | 0.25 |  |
| 1024 | 512 | 64 | 64 | 0.0625 | 0.125 | 0.1875 |  |
| 1024 | 512 | 32 | 64 | 0.03125 | 0.125 | 0.15625 |  |
| 1024 | 512 | 16 | 128 | 0.015625 | 0.25 | 0.265625 |  |
| 1024 | 512 | 8 | 256 | 0.0078125 | 0.5 | 0.5078125 |  |
| 1024 | 512 | 8 | 512 | 0.0078125 | 1 | 1.0078125 |  |
| 1024 | 512 | 0 | 512 | 0 | 1 | 1 |  |

Abbreviations: CST, colistin; TGC, tigecycline; VAN, vancomycin; RIF, rifampin; IMP, Imipenem AMK, amikacin; ATM, aztreonam; CAZ, ceftazidime. MIC, minimum inhibitory concentration; FIC, fractional inhibitory concentration; FICI, fractional inhibitory concentration index.
